# Clinical significance of neutrophil extracellular traps biomarkers in thrombosis

**DOI:** 10.1186/s12959-022-00421-y

**Published:** 2022-10-12

**Authors:** Xiangbo Xu, Yuting Wu, Shixue Xu, Yue Yin, Walter Ageno, Valerio De Stefano, Qingchun Zhao, Xingshun Qi

**Affiliations:** 1 Department of Gastroenterology, General Hospital of Northern Theater Command (the Teaching School of Shenyang Pharmaceutical University), Shenyang, China; 2 Department of Life Science and Biochemistry, Shenyang Pharmaceutical University, Shenyang, China; 3 Department of Pharmacy, General Hospital of Northern Theater Command (the Teaching School of Shenyang Pharmaceutical University), Shenyang, China; 4 Department of Medicine and Surgery, University of Insubria, Varese, Italy; 5 Department of Radiological and Hematological Sciences, Catholic University, Fondazione Policlinico A. Gemelli IRCCS, Section of Hematology, Rome, Italy

**Keywords:** Neutrophil, Neutrophil extracellular traps, Thrombosis, Citrullinated histones, Myeloperoxidase

## Abstract

Neutrophil extracellular traps (NETs) may be associated with the development of thrombosis. Experimental studies have confirmed the presence of NETs in thrombi specimens and potential role of NETs in the mechanisms of thrombosis. Clinical studies also have demonstrated significant changes in the levels of serum or plasma NETs biomarkers, such as citrullinated histones, myeloperoxidase, neutrophil elastase, nucleosomes, DNA, and their complexes in patients with thrombosis. This paper aims to comprehensively review the currently available evidence regarding the change in the levels of NETs biomarkers in patients with thrombosis, summarize the role of NETs and its biomarkers in the development and prognostic assessment of venous thromboembolism, coronary artery diseases, ischemic stroke, cancer-associated thromboembolism, and coronavirus disease 2019-associated thromboembolism, explore the potential therapeutic implications of NETs, and further discuss the shortcomings of existing NETs biomarkers in serum and plasma and their detection methods.

## Introduction

Thrombosis, which refers to the formation of blood clots in arterial and venous vessels, is a consequence of inherited or acquired imbalance of procoagulant, anticoagulant, and fibrinolytic factors [1], and results in high morbidity and mortality [2, 3]. Knowledge regarding underlying mechanisms of thrombosis is necessary to improve its management strategy. Traditionally, it is thought that thrombus should be formed by the interaction of platelets, fibrin, and red blood cells. Neutrophils are the first-line defense against invading pathogens [4]. Recently, it has been recognized that the release of neutrophil extracellular traps (NETs) may contribute to the development of thrombosis [5–8]. NETs release is caused by stimulated neutrophils which form web-like structures mainly composed of extracellular DNA, histones, and granular proteins, such as neutrophil elastase (NE), myeloperoxidase (MPO), and calprotectin, etc [9, 10]. The current review paper primarily aims to summarize the role of NETs and its biomarkers in the development and prognostic assessment of venous thromboembolism (VTE), coronary artery diseases (CAD), ischemic stroke (IS), cancer-associated thromboembolism, and coronavirus disease 2019 (COVID-19)-associated thromboembolism, explore the potential therapeutic implications of NETs, and further discuss the shortcomings of existing NETs biomarkers in serum and plasma and their detection methods.

### Mechanisms of NETs formation

**Fig. 1:**
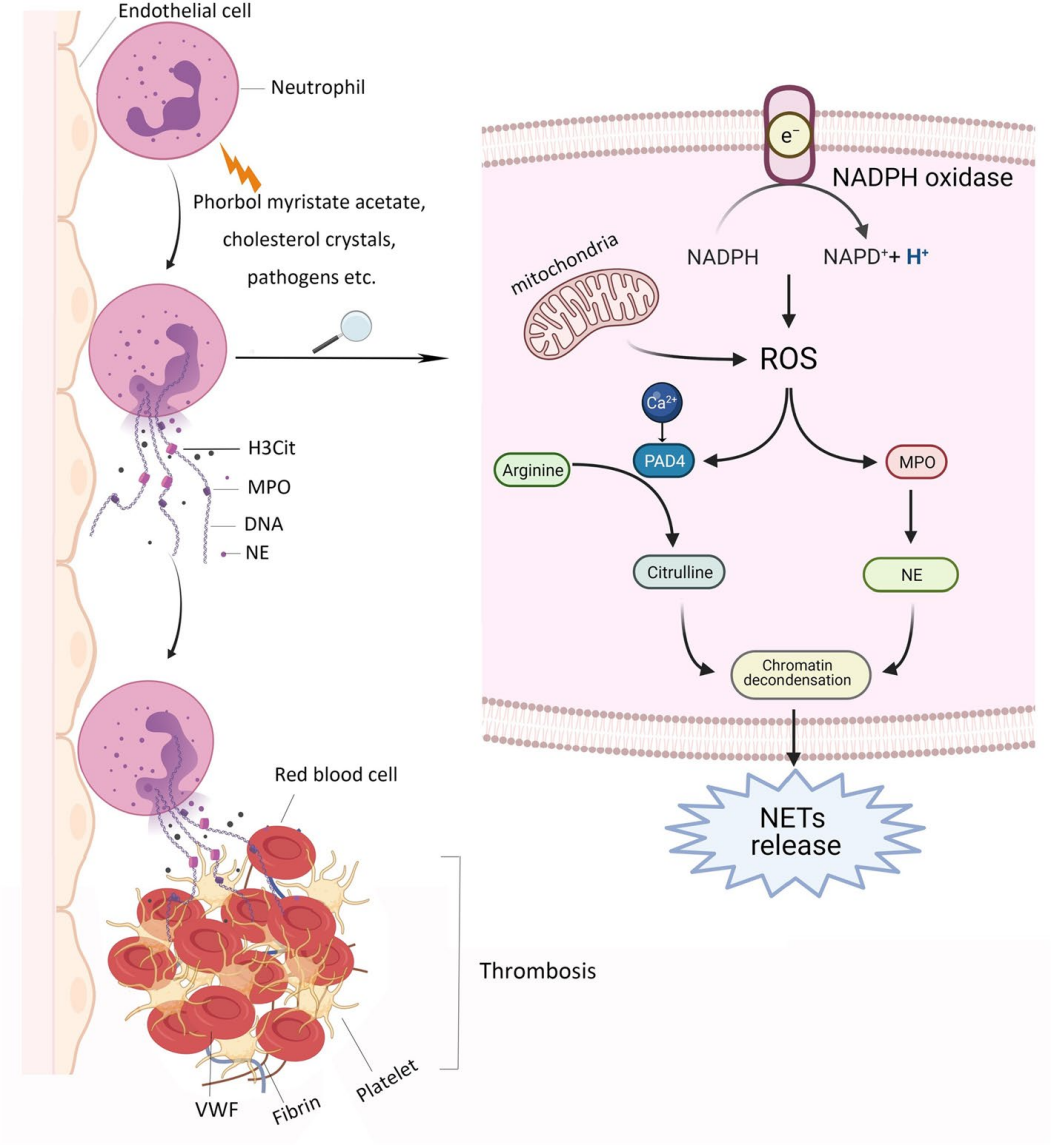
NETs formation and thrombosis ***Abbreviations:*** H3Cit, Citrullinated histone H3; MPO, Myeloperoxidase; NADPH, Nicotinamide adenine dinucleotide phosphate; NE, Neutrophil elastase; NETs, Neutrophil extracellular traps; PAD4, Peptidyl arginine deiminase 4; ROS, Reactive oxygen species

NETs formation, a unique form of cell death process [11], release decondensed chromatin and granular proteins with nuclear materials [12]. Until now, there are two potential mechanisms of NETs formation [13]. The first mechanism is lytic-NETs formation, which can be induced by phorbol myristate acetate or cholesterol crystal. Peptidyl arginine deiminase 4 (PAD4) may be activated by reactive oxygen species (ROS) [13–15], which can be generated by nicotinamide adenine dinucleotide phosphate (NADPH) or mitochondria [16, 17], or calcium ionophore [18], thereby leading to the citrullination of arginine residues of histones [18]. Notably, gasdermin D is required for ROS generation [19]. Meanwhile, MPO and NE can be translocated by ROS into the nucleus [20]. Subsequently, neutrophils exhibit rapid disassembly of the actin cytoskeleton, followed by shedding of plasma membrane microvesicles, disassembly and remodeling of the microtubule and vimentin cytoskeletons, endoplasmic reticulum vesiculation, chromatin decondensation and nuclear rounding, and progressive permeabilization of plasma membrane and nuclear envelope [21]. Then, protein kinase C α-mediated lamin B phosphorylation drives nuclear envelope rupture to release chromatin [22]. Finally, NETs are released after plasma membrane rupture [21] (Fig. 1). The second mechanism is non-lytic NETs formation, which can be induced by certain bacteria, such as *E. coli*, *S aureus*, or *Candida albicans*, through the activation of neutrophils mediated by Toll-like receptors (TLRs) or complement receptors [23], independent of NADPH oxidase activation. By this way, neutrophils are still alive and preserve their functions to move and phagocytose to some extent [23]. Besides, autophagy may provide another insight into the mechanisms of NETs formation [24, 25]. Collectively, some biomarkers involved in the NETs formation should include independent extracellular DNA, proteins derived from neutrophils (i.e., MPO and NE), proteins required for NETs formation (i.e., PAD4 and citrullinated histones), and their complexes.

### NETs promote thrombosis

NETs may contribute to the development of thrombosis by forming a “scaffold”, which induces platelets adhesion, activation, and aggregation, recruits red blood cells, and maintains the stability of thrombus together with fibronectin, fibrinogen, and von Willebrand factor (VWF) [26]. The interaction of neutrophils with platelets depends on the adhesion molecules, such as P-selectin, P-selectin glycoprotein ligand 1, glycoprotein Ib, and macrophage-1 antigen [27]. Additionally, platelet-derived high mobility group box 1 (HMGB1) mediates both NETs formation and thrombosis [28, 29]. HMGB1 can interact with TLR4 [30], enabling neutrophils to release NETs. Furthermore, HMGB1 can promote early recruitment of platelets [31], thereby enhancing the pro-thrombotic effect and promoting the development of thrombosis.

The components of NETs themselves can also affect the formation of thrombosis. Histones are responsible for tissue factor activity [32], platelet activation via mediating TLR2 and TLR4 [33], platelet aggregation via inducing calcium influx and fibrinogen recruitment [34], reduction of thrombomodulin-dependent protein C activation [35], and release of activated thrombin [36, 37]. Furthermore, histone 4 promotes prothrombin autoactivation to thrombin [38]. DNA, which is deemed as another component of NETs, is reported to shorten clotting time, promote FXII activation and FXIa generation, and amplify tissue factor-initiated thrombin generation [12]. Both histones and DNA can increase the median fiber diameter of plasma clots [39]. PAD4 can accelerate the development of thrombosis via protecting VWF-platelets string from the cleavage of endogenous a disintegrin and metalloproteinase with thrombospondin type-1 motif-13 (ADAMTS13) [40]. Other components of NETs, including NE, cathepsin G, and nucleosomes, are responsible for promoting coagulation and intravascular thrombus growth through enhancing intrinsic and extrinsic coagulation pathways [41].

Collectively, NETs can affect the development of thrombosis via multiple ways. Additional evidence regarding how NETs promote thrombosis is also emerging.

### NETs biomarkers and VTE

VTE primarily comprises of deep vein thrombosis (DVT) and pulmonary embolism (PE). Experimental and clinical studies have confirmed the presence of NETs biomarkers in VTE specimens. Extracellular DNA was in close proximity to neutrophils, together with positive staining of MPO, NE, and histones by immunostaining assay after induction of venous thrombosis [42]. Additionally, citrullinated histone H3 (H3Cit) was observed in the red [43] or fresh red fibrin-rich parts of thrombi [44]. In baboons with iliac vein thrombosis, dotted and diffuse staining of DNA and positive staining of DNA-histone could be observed in thrombi [26]. Human venous thrombi from surgical samples or autopsies revealed the colocalization of DNA, DNA-histone complexes, and MPO [45], that of DNA, MPO, CD11b, and H3Cit [46], and that of DNA, MPO, H3Cit, pan-Cit, and PAD4 in organizing thrombi [46].

NETs biomarkers have been quantitatively evaluated in VTE patients in several clinical studies [44, 47–58] (Table 1). The levels of plasma DNA [53], H3Cit-DNA [58], and NE [58] were elevated in VTE patients. The level of plasma MPO had a good diagnostic performance of VTE [59], while the diagnostic accuracy of H3Cit-DNA and NE was not superior to that of D-dimer [58]. On the other hand, the expression of NETs biomarkers may depend on the locations of VTE. The levels of plasma DNA and nucleosomes were significantly different between elderly patients with PE and DVT [50]. Besides, the levels of plasma DNA and calprotectin were higher in patients with splanchnic vein thrombosis (SVT) than those with DVT, whereas the level of MPO was much higher in patients with DVT of the lower limbs than those with SVT [52]. Clinical evidence regarding NETs biomarkers in patients with VTE at various locations are separately reviewed in the following paragraphs.

**Table 1:** Studies evaluating NETs biomarkers in VTE

First author/year	Study design	Included patients	Groups (No. patients)	Samples processing	NETs biomarkers	Analytical methods for NETs biomarkers	Detailed values
Arnalich et al (2013) [47]	Case–control and cohort	Patients with acute massive or sub-massive PE, confirmed with computed tomographic pulmonary angiography	Massive PE (*n* = 37) vs. Sub-massive PE (*n* = 37) vs. HC (*n* = 37)	Plasma, 4 ºC, 1800 × g,10 min	Mitochondrial DNA	qPCR	2970 vs. 870 vs. 185 GE/mL
					Nuclear DNA	qPCR	3325 vs. 1245 vs. 520 GE/mL
Diaz et al (2013) [48]	Case–control	Patients performed duplex ultrasound to confirm the presence of DVT	DVT (*n* = 47) vs. Negative DVT (*n* = 28) vs. HC (*n* = 19)	Plasma, 4 °C, 2000 × g, 10 min	MPO	ELISA	31.7 vs. 15.5 vs. 5.7 AU
					DNA	SytoxGreen fluorimetry	57.7 vs. 17.9 vs. 23.9 ng/mL
van Montfoort et al (2013) [49]	Case–control	Adult patients with and without acute symptomatic DVT of the leg	DVT (*n* = 150) vs. No DVT (*n* = 195)	Plasma, RT, 1500 × g, 15 min	NE-α1-antitrypsin	ELISA	53 vs. 45 ng/mL
					Nucleosomes	ELISA	17 vs. 9 U/mL
Jiménez-Alcázar et al (2018) [50]	Case–control and cohort	Patients aged > 65 years with acute, symptomatic VTE	Distal DVT (*n* = 51) vs. Proximal DVT (*n* = 133) vs. PE (*n* = 427)	Plasma	DNA-histone-MPO	ELISA	NA
					Nucleosomes	ELISA	NA
					DNA	SytoxGreen fluorimetry	NA
Lee et al (2018) [51]	Case–control	Patients with sepsis and thrombosis	DVT (*n* = 25) vs. HC (*n* = 23)	Serum, 4 °C, 1500 × g, 15 min	MPO	ELISA	250.5 vs. 120.4 ng/mL
					MPO-DNA	ELISA	0.07 vs. 0.05 OD
					Nucleosomes	ELISA	0.3 vs. 0.1 U/L
					NE	ELISA	370.8 vs. 162.4 ng/mL
					DNA	SytoxGreen fluorimetry	22.3 vs.8.1 ng/mL
Martos et al (2020) [52]	Case–control	Patients with VTE	DVT (*n* = 192) vs. SVT (*n* = 61) vs. HC (*n* = 249)	Plasma, 4 ℃, 1811 × g, 30 min	MPO	ELISA	1728.5 vs. 1882.5 vs. 1250.0 ng/mL
					DNA	PicoGreen fluorimetry	1657.6 vs. 1586.4 vs. 1320.9 ng/mL
					Calprotectin	ELISA	NA
Medeiros et al (2020) [53]	Case–control	Patients with VTE and anticoagulation therapy	VTE off warfarin (*n* = 263) vs. VTE on warfarin (*n* = 245) vs. HC (*n* = 50)	Plasma, 20 °C, 1700 × g, 15 + 5 min	DNA	QIAamp	5.53 vs. 3.11 vs. 2.77 µg/mL
Ząbczyk et al (2020) [54]	Case–control and cohort	Patients with acute PE	Acute PE (*n* = 126) vs. HC (*n* = 25)	Plasma, 2500 × g, 10 min	H3Cit	ELISA	2.77 vs. 0.59 ng/mL
Liu et al (2021) [55]	Case–control	Patients with traumatic fracture	Trauma non-DVT (*n* = 37) vs. Trauma DVT (*n* = 39) vs. DVT (*n* = 34) vs. HC (*n* = 24)	Plasma, 2500 × g, 15 min	H3Cit	ELISA	0.38 vs. 0.87 vs. 1.88 vs. 1.79 ng/mL
					Nucleosomes	ELISA	1.20 vs. 1.03 vs. 1.29 vs.—ratio
					DNA	PicoGreen fluorimetry	185.56 vs. 165.70 vs. 216.15 vs. 135.08 ng/mL
Sharma et al (2021) [44]	Case–control	Patients with stable CTEPH	CTEPH (*n* = 141) vs. Controls (*n* = 60)	Plasma, 2000 × g, 10 min	MPO	ELISA	NA
					H3Cit	ELISA	NA
					DNA	SytoxGreen fluorimetry	NA
Turon et al (2021) [56]	Cohort	Patients with cirrhosis	PVT (*n* = 23) vs. No PVT (*n* = 287)	Plasma	MPO-DNA	ELISA	0.21 vs. 0.29 AU
					DNA	PicoGreen fluorimetry	0.89 vs. 0.89 µg/mL
Xing et al (2022) [57]	Case–control	Patients with cirrhosis	PVT (*n* = 28) vs. No PVT (*n* = 44)	Plasma, 1000 × g, 15 min	MPO	ELISA	NA
					NE	ELISA	NA
					H3Cit	ELISA	NA
Smith et al (2022) [58]	Case–control	Patients with VTE	VEBIOS ER Cohort: VTE (*n* = 51) vs. No VTE (*n* = 96) vs. HC (*n* = 30) DFW-VTE Cohort: VTE (*n* = 61) vs. No VTE (*n* = 86) vs. HC (*n* = 30)	Plasma, 3000 × g, 15 min	H3Cit-DNA	ELISA	VEBIOS ER Cohort: 110 vs. 73 vs. 38 ng/mL DFW-VTE Cohort: 102 vs. 54 vs. 38 ng/mL
					NE	ELISA	VEBIOS ER Cohort: 31 vs. 24 vs.21 ng/mL DFW-VTE Cohort: 49 vs. 38 vs. 21 ng/mL
					DNA	PicoGreen fluorimetry	VEBIOS ER Cohort: 423 vs. 405 vs. 421 ng/mL DFW-VTE Cohort: 396 vs. 392 vs. 421 ng/mL

*Abbreviations:* *AU* Absorbance unit, *CTEPH* Chronic thromboembolic pulmonary hypertension, *DVT* Deep vein thrombosis, *HC* Healthy control, *H3Cit* Citrullinated histone H3, *ELISA* Enzyme-linked immunosorbent assay, *Min* Minute, *MPO* Myeloperoxidase, *NA* Not available, *NE* Neutrophil elastase, *NETs* Neutrophil extracellular traps, *OD* Optical density, *PE* Pulmonary embolism, *PVT* Portal vein thrombosis, *qPCR* Quantitative polymerase chain reaction, *RT* Room temperature, *SVT* Splanchnic vein thrombosis, *VTE* Venous thromboembolism

### *DVT*

In a case–control study, the levels of plasma NE-α1-antitrypsin complexes and nucleosomes ≥ 80th percentile (odds ratio [OR] = 3.0 and OR = 2.4) significantly increased the risk of symptomatic DVT regardless of adjustment for potential confounders [49]. By contrast, another study did not show any significant difference in the levels of serum NE and nucleosomes between patients with DVT and healthy controls [51]. Thus, more evidence is necessary to clarify the association of NE and nucleosomes with DVT. Notably, among the currently published studies, the levels of serum MPO, MPO-DNA, and DNA were significantly higher in patients with DVT than those without DVT or healthy controls [48, 51, 52]. Furthermore, the levels of plasma H3Cit and DNA can be used for the diagnosis of DVT in patients with traumatic fractures [55].

### *PE*

A recent study found that the levels of plasma neutrophils, MPO, and DNA, rather than H3Cit, were significantly elevated in patients with chronic thromboembolic pulmonary hypertension as compared with healthy controls [44]. By contrast, another study demonstrated that the level of plasma H3Cit was almost fivefold higher in patients with acute PE than healthy controls [54]. Such a difference in the expression of plasma H3Cit between the two studies might be attributed to the stage of disease (chronic versus acute). On the other hand, NETs biomarkers can also reflect the severity of PE. The levels of plasma DNA deriving from mitochondria and nucleus were higher in patients with massive PE than those with sub-massive PE [47]. Notably, it should be acknowledged that this change of NETs biomarkers might also be derived from damaged tissues during severe PE. Additionally, higher level of plasma DNA was independently associated with increased PE-related mortality [50] and all-cause mortality [47, 50], but not the recurrence of VTE during a 3-year follow-up period [50]. Similarly, the level of plasma H3Cit could also predict acute PE-related death [54].

### *PVT*

A European prospective cohort study did not find any significant relationship between the levels of plasma MPO-DNA and DNA at baseline and the development of portal vein thrombosis (PVT) in patients with liver cirrhosis during a mean follow-up period of 48 months [56]. However, it should be noted that a majority of patients included in this cohort study had Child–Pugh class A, suggesting that they had well-preserved hepatic function [60]. By comparison, a Chinese cross-sectional study, in which a majority of cirrhotic patients included had Child–Pugh class B + C (69.4%), demonstrated that the levels of plasma H3Cit, NE, and MPO were significantly higher in patients with PVT than those without PVT, and positively correlated with thrombin-antithrombin (TAT) complex and FX, which are well-known markers for hypercoagulability [57]. Such a controversy should be further clarified in cirrhotic patients according to the severity of liver dysfunction.

### NETs biomarkers and CAD

CAD encompasses stable angina, unstable angina, myocardial infarction (MI), and sudden cardiac death due to the occurrence of atherosclerosis or thrombosis in coronary arteries [61]. NETs formation has been detected by positive staining of Ly6G, DNA, MPO, and H3Cit in mice’s atherosclerotic lesions [62–64]. Immunostaining assay found the colocalization of CD177, NE, and DNA in patients’ carotid plaques [65]. By immunostaining of patients’ carotid and coronary plaques, another study also demonstrated that CD66b, NE, H4Cit, and DNA were in contact with the luminal surface of erosion-prone plaques and localized within rupture-prone plaques [66]. Additionally, the colocalization of histones, NE, and MPO was commonly observed in fresh and lytic coronary thrombi from MI patients, rather than organized coronary thrombi [67]. The other colocalizations of MPO, H3Cit, and DNA [68] and DNA, DNA-histone complexes, and MPO [45] were also detected in coronary thrombi.

Some clinical studies have been performed to evaluate the importance of NETs biomarkers in CAD patients [69–80] (Table 2). It seems that NETs biomarkers could predict the disease severity, hypercoagulability, and worse clinical outcomes in CAD patients. The levels of plasma MPO-DNA, nucleosomes, and DNA were significantly elevated in patients with more severe CAD, and could predict the number of diseased coronary artery segments and the incidence of major adverse cardiac events (MACE). Among them, only higher level of plasma nucleosomes was an independent risk factor for severe coronary stenosis, and only higher level of plasma DNA was independently associated with prothrombotic state [71]. Another large-scale study involving 1001 CAD patients found that higher level of serum DNA was significantly associated with hypercoagulability and predicted worse clinical outcomes [73]. Both studies suggested that DNA could predict hypercoagulability, and other NETs biomarkers, such as nucleosomes and MPO-DNA, might be useful to predict CAD progression.

**Table 2:** Studies evaluating NETs biomarkers in CAD

First author/year	Study design	Included patients	Groups (No. patients)	Samples processing	NETs biomarkers	Analytical methods for NETs biomarkers	Detailed values
Antonatos et al (2006) [69]	Case–control and cohort	Patients with acute MI and underwent thrombolysis with reteplase within 6 h from onset of pain	Acute MI (*n* = 13) vs. HC (*n* = 30)	Plasma, 800 × g and 16,000 × g	DNA	qPCR	6873 vs. 4112 GE/mL
Shimony et al (2010) [70]	Case–control	Patients with acute STEMI	STEMI (*n* = 16) vs. HC (*n* = 47)	Serum	DNA	Sybr Gold fluorimetry	747 vs. 471 ng/mL
Borissoff et al (2013) [71]	Case–control and cohort	Patients with chest discomfort symptoms, suspected for CAD	Extremely calcified (*n* = 37) vs. Severe CAD (*n* = 45) vs. Moderate CAD (*n* = 74) vs. Mild CAD (*n* = 75) vs. No CAD (*n* = 51)	Plasma, 2000 × g, 15 min, 11,000 × g, 10 min	MPO-DNA	ELISA	NA
					Nucleosomes	ELISA	NA
					DNA	SytoxGreen fluorimetry	79.37 (Extremely calcified) vs. 69.59 (Severe CAD) vs. 50.09 (No CAD) ng/mL
Cui et al (2013) [72]	Case–control	Patients with ACS and SA controls	ACS (*n* = 137) vs. SA (*n* = 13) vs. HC (*n* = 45)	Plasma, 25 °C, 1600 × g, 10 min, 16,000 × g, 1 min	DNA	Alu sequence-based bDNA assay	2285.0 vs. 202.3 vs. 118.3 ng/mL
Ramirez et al (2016) [79]	Case–control	Patients with STEMI underwent PCI within 1–6 h from the onset of chest pain and chronic SA controls	STEMI vs. Chronic SA vs. HC	Plasma, 4 °C, 320 × g, 15 min, 100,000 × g, 5 min	H4Cit	ELISA	NA
					MPO-DNA	ELISA	NA
Langseth et al (2018) [73]	Cohort	Patients with angiographically verified CAD, on aspirin monotherapy for at least 1 w	Clinical endpoint (*n* = 402) vs. No clinical endpoints (*n* = 394)	Serum, 2500 × g, 10 min	MPO-DNA	ELISA	NA
					DNA	PicoGreen fluorimetry	402 vs. 394 ng/mL
Helseth et al (2019) [74]	Cohort	Patients with first-time STEMI within 6 h of symptom onset admitted for PCI	Before PCI (*n* = 259) vs. After PCI vs. (*n* = 258) vs. After PCI 1 d (*n* = 251/254) vs. After PCI 4 m (*n* = 258)	Serum, 2500 × g, 10 min	MPO-DNA	ELISA	NA
					DNA	PicoGreen fluorimetry	NA
Lim et al (2019) [75]	Case–control	Patients with newly diagnosed ACS or AIS	ACS (*n* = 37) vs. AIS (*n* = 58) vs. HC (*n* = 25)	Plasma, 1600 × g, 15 min	DNA-histone	ELISA	19.73 vs. 13.71 vs. 14.32 mU
					DNA	PicoGreen fluorimetry	743.28 vs. 524.22 vs. 216.48 ng/mL
Liu et al (2019) [76]	Cohort	Patient was enrolled within 12 h of the onset of clinical signs and had STEMI with TIMI flow 0 before emergent PCI	Infarct-related artery (*n* = 36) vs. Peripheral arteries (*n* = 36)	Plasma	MPO-DNA	ELISA	0.44 vs. 0.28
					DNA	SytoxGreen fluorimetry	0.41 vs.0.31 µg/mL
Hofbauer et al (2019) [77]	Cohort and case–control	Patients with STEMI undergoing primary PCI for a coronary TIMI flow of 0	Culprit site (*n* = 48) vs. Femoral site (*n* = 48) vs. HC (*n* = 21)	Plasma, 1000 × g, 10 min	H3Cit	ELISA	332 vs. 235 vs. 192 ng/mL
					DNA	PicoGreen fluorimetry	529 vs. 404 vs. 291 ng/mL
Langseth et al (2020) [78]	Cohort	Patients diagnosed with STEMI admitted for PCI	Anterior MI (*n* = 413) vs. Other locations of infraction (*n* = 543)	Serum, 2500 × g, 10 min	H3Cit	ELISA	9.71 vs. 8.69 ng/mL
					MPO-DNA	ELISA	0.188 vs. 0.171 OD
					DNA	PicoGreen fluorimetry	424 vs. 409 ng/mL
Hally et al (2021) [80]	Case–control	Patients diagnosed with MACE post-AMI within 1-year follow-up period	MACE (*n* = 100) vs. No MACE (*n* = 200)	Serum, 1500 × g, 12 min	MPO-DNA	ELISA	5.09 vs. 4.67 (% of NETs standard)
					NE-DNA	ELISA	2.05 vs. 1.97 (% of pooled serum standard)
					H3Cit	ELISA	7.07 vs. 5.44 (% of NETs standard)

*Abbreviations:* *ACS* Acute coronary syndrome, *AIS* Acute ischemic stroke, *AMI* Acute myocardial infarction, *CAD* Coronary artery disease, *D* Day, *ELISA* Enzyme-linked immunosorbent assay, *H* Hour, *H3Cit* Citrullinated histone H3, *HC* Healthy control, *M* Month, *MACE* Major adverse cardiovascular events, *MI* Myocardial infarction, *Min* Minute, *MPO* Myeloperoxidase, *NA* Not available, *NE* Neutrophil elastase, *NETs* Neutrophil extracellular traps, *OD* Optical density, *PCI* Percutaneous coronary intervention, *qPCR* Quantitative polymerase chain reaction, *SA* Stable angina, *STEMI* ST-segment elevation myocardial infarction, *TIMI* Thrombolysis in myocardial infarction, *W* Week

### *MI*

MI is primarily associated with plaque rupture and erosion [81]. Until now, the role of NETs biomarkers in patients with MI has been more comprehensively explored as compared to those with other types of CAD. The level of plasma DNA was higher in patients with acute MI (AMI) than healthy controls [69] and stable angina [72], and positively correlated with Gensini and GRACE scores [72] and peak levels of creatine kinase (CK) and troponin-T [70]. Particularly, in ST elevation MI (STEMI) patients admitted for percutaneous coronary intervention (PCI), the levels of plasma H3Cit [77], MPO-DNA [76, 78], and DNA [76–78] were significantly higher in infarct-related coronary arteries than peripheral arteries [76, 77] or in anterior MI than other locations of infarction [78]. The levels of serum MPO-DNA and DNA became the highest in STEMI patients before PCI, and decreased after PCI [74]. Both H3Cit and DNA levels positively correlated with infarct size [74, 77], and high level of DNA was usually associated with increased risk of developing lower left ventricular ejection fraction [74], adverse clinical events [76], and all-cause mortality [78]. Importantly, DNA level had a predictive value for in-hospital mortality in STEMI patients, which was equivalent to that of troponin I [75], troponin T, and CK-MB [76]. Higher level of serum DNA is also associated with hypercoagulability indicated by elevated D-dimer and prothrombin fragment 1 + 2 levels in STEMI patients [78]. A composite score of NETs biomarkers and platelet count showed the most favorable predictive value for MACE in non-ST and STEMI patients [80].

### NETs biomarkers and IS

IS can be caused by cardiac embolism, atherosclerosis of cerebral circulation, and occlusion of small vessels resulting in high mortality and disability worldwide [82]. In a rat model, a significant increase in the level of serum DNA was observed at 24 h after the onset of IS. DNA level was positively associated with the total infarct volume, brain edema, and neurologic severity score (correlation coefficient = 0.78, 0.91, and 0.73, respectively) [83]. Abundant neutrophils and NETs were also found in thrombi from patients with acute IS (AIS) by the colocalization of CD66b, H3Cit, and DNA, that of H3Cit and NE [84], or that of H4Cit, MPO, and DNA [85]. Meanwhile, neutrophils and H3Cit were especially higher in older thrombi than fresh thrombi by calculating the area of H3Cit positive staining [84].

NETs biomarkers have been explored in AIS patients (Table 3) [75, 86, 87]. In a prospective cohort study, the level of plasma DNA was elevated by threefold in AIS patients compared with non-AIS patients, and exhibited a positive correlation with infarct size [86]. Besides, the levels of plasma nucleosomes and H3Cit were also elevated in AIS patients with a history of atrial fibrillation, NIHSS score > 14 at onset, NIHSS score ≥ 6 at discharge, and mRankin scale score > 2 at discharge [87]. The highest quartile level of plasma H3Cit was independently associated with atrial fibrillation (OR = 6.7) and all-cause mortality (OR = 7.1) during one-year follow-up period [87]. Furthermore, the levels of plasma H3Cit, MPO, and DNA were significantly increased in IS patients with elevated hypersensitive troponin T levels as compared to those with normal hypersensitive troponin T levels [88].

**Table 3:** Studies evaluating NETs biomarkers in IS

First author/year	Study design	Included patients	Groups (No. patients)	Samples processing	NETs biomarkers	Analytical methods for NETs biomarkers	Detailed values
O'Connell et al (2017) [86]	Case–control	Patients experiencing AIS and those identified as stroke mimics	AIS (*n* = 43) vs. Negative AIS (*n* = 20)	Plasma, 2000 × g, 10 min and 10,000 × g, 10 min	DNA	qPCR	NA
Vallés et al (2017) [87]	Case–control and cohort	Patients with AIS during the acute phase of brain ischemia and suffering stroke < 24 h before admission	AIS (*n* = 243) vs. HC (*n* = 27)	Plasma, 22 °C, 2500 × g, 10 min	H3Cit	ELISA	0.080 vs. 0.039 AU
					Nucleosomes	ELISA	0.329 vs. 0.209 AU
					DNA	SytoxGreen fluorimetry	432.11 vs. 324.2 ng/mL
Lim et al (2020) [75]	Case–control and cohort	Patients with newly diagnosed ACS or AIS	ACS (*n* = 37) vs. AIS (*n* = 58) vs. HC (*n* = 25)	Plasma, 1600 × g, 15 min	DNA-histone	ELISA	19.73 vs. 13.71 vs. 14.32 mU
					DNA	PicoGreen fluorimetry	743.28 vs. 524.22 vs. 216.48 ng/mL

*Abbreviations:* *ACS* Acute coronary syndrome, *AIS* Acute ischemic stroke, *AU* Absorbance unit, *H3Cit* Citrullinated histone H3, *ELISA* Enzyme-linked immunosorbent assay, *HC* Healthy control, *IS* Ischemic stroke, *Min* Minute, *NA* Not available, *NETs* Neutrophil extracellular traps, *qPCR* Quantitative polymerase chain reaction

### NETs biomarkers and cancer-associated thromboembolism

Thromboembolism is one of the most common comorbidities associated with cancer and also a leading cause of death for cancer patients [89, 90]. NETs formation has been detected in animal models of cancer and patients with cancer-associated thrombosis. Increased levels of plasma H3Cit, NE, and DNA were found in mice bearing pancreatic tumors [91]. Additionally, in murine models of chronic myelogenous leukemia and breast and lung cancers, NETs formation was implied by the colocalization of DNA, fibrin, and VWF in thrombi as well as web-like patterns [92]. In patients with gastric cancer, the levels of NETs biomarkers released by neutrophils cultured in vitro were positively associated with TAT complex and D-dimer levels, indicating that NETs might contribute to hypercoagulability [93]. Besides, in patients with cancer, NETs formation was indicated by the colocalization of H3Cit and DNA in cerebral, coronary, and pulmonary microthrombi [88].

Recently, clinical studies have focused on the association between NETs biomarkers and cancer-associated thromboembolism[88, 94–98] (Table 4).

**Table 4:** Studies evaluating NETs biomarkers in cancer-associated thromboembolism

First author/year	Study design	Included patients	Groups (No. patients)	Samples processing	NETs biomarkers	Analytical methods for NETs biomarkers	Detailed values
Thålin et al (2016) [88]	Case–control	Patients with IS	Cancers (*n* = 8) vs. No cancers (*n* = 23)	Plasma, 2000 × g, 20 min	H3Cit	ELISA	0.22 vs. 0.07 OD
					MPO	ELISA	74.1 vs. 37.8 ng/mL
					DNA	PicoGreen fluorimetry	504.0 vs. 407.9 ng/mL
Mauracher et al (2018) [94]	Cohort	Adult patients with newly diagnosed malignancy or progression of disease after remission	VTE (*n* = 89) vs. No VTE (*n* = 857)	Plasma, 3000 × g, 10 min	H3Cit	ELISA	52.4 vs. 24.1 ng/mL
					Nucleosomes	ELISA	1.3 vs. 1.2 MoM
					DNA	PicoGreen fluorimetry	384.5 vs. 355.8 ng/mL
Bang et al (2019) [95]	Case–control	Patients with active cancer	Cancer-stroke (*n* = 38) vs. Stroke-control (*n* = 40) vs. Cancer-control (*n* = 27) vs. HC (*n* = 33)	Plasma, 2000 × g, 15 min	Nucleosomes	ELISA	0.379 vs. 0.189 vs. 0.251 vs. 0.194 OD
					DNA	PicoGreen fluorimetry	40.35 vs. 34.38 vs. 34.52 vs. 30.48 mg/mL
Grilz et al (2019) [96]	Cohort	Adult patients with newly diagnosed malignancy or a progression of disease after complete or partial remission	ATE (*n* = 22) vs. No ATE (*n* = 935)	Plasma, 3000 × g, 10 min	H3Cit	ELISA	NA
					Nucleosomes	ELISA	NA
					DNA	PicoGreen fluorimetry	NA
Guy et al (2019) [98]	Case–control	Patients with MPN	Thrombosis (*n* = 16) vs. No thrombosis (*n* = 15)	Plasma, 2400 × g, 15 min	DNA	PicoGreen fluorimetry	NA
					MPO-DNA	ELISA	NA
Seo et al (2019) [97]	Case–control	Patients with HCC	PVT (*n* = 77) vs. No PVT (*n* = 100)	Plasma, 1550 × g, 15 min	DNA-histone	ELISA	159 vs. 83 AU
					NE	ELISA	NA
					DNA	PicoGreen fluorimetry	142.1 vs. 127.0 ng/mL

*Abbreviations:* *ATE* Arterial thromboembolism, *AU *Absorbance unit, *ELISA* Enzyme-linked immunosorbent assay, *H3Cit* Citrullinated histone H3, *HC* Healthy control, *HCC* Hepatocellular carcinoma, *IS* Ischemic stroke, *Min* Minute, *MPN* Myeloproliferative neoplasms, *MPO* Myeloperoxidase, *NA* Not available, *NE* Neutrophil elastase, *NETs* Neutrophil extracellular traps, *OD* Optical density, *PVT* Portal vein thrombosis, *VTE* Venous thromboembolism

### *VTE*

The level of plasma nucleosomes was an independent risk factor for DVT, irrespective of malignancy [49]. However, in a large-scale study of 946 patients with malignancy, higher levels of plasma nucleosomes and DNA could only predict a higher risk of VTE, including PE, DVT, and SVT, during the first 6-month follow-up period, but only higher level of plasma H3Cit was an independent predictor of VTE during the overall follow-up period and comparable to D-dimer, soluble P-selectin, FVIII, and prothrombin fragment 1 + 2 for predicting VTE. More importantly, H3Cit significantly increased the risk of VTE in patients with pancreatic and lung cancer, but not those with cancers in other sites [94]. The levels of plasma DNA-histone and DNA, rather than NE, were significantly higher in hepatocellular carcinoma patients with PVT than those without PVT[97]. In patients with colorectal cancer, the levels of plasma MPO-DNA and DNA also positively correlated with the levels of plasma TAT complex and D-dimer, suggesting that NETs may contribute to coagulation activation and increased risk of VTE [99].

#### *Arterial thrombosis*

The levels of plasma nucleosomes and DNA were significantly elevated in cancer-related stroke patients compared with healthy-, cancer-, and stroke-controls. High plasma DNA level was independently associated with the risk of cancer-related stroke [95]. Furthermore, the levels of plasma H3Cit, MPO, and DNA were significantly elevated in IS patients with cancer as compared to those without [88]. Conversely, a prospective observational cohort study revealed that the levels of plasma H3Cit, DNA, and nucleosomes at baseline could not predict a composite outcome of MI, IS, and peripheral arterial occlusion in patients with malignancy, although H3Cit and DNA significantly increased the risk of death [96]. The level of plasma MPO-DNA was higher in myeloproliferative neoplasms (MPNs) patients with a history of arterial and venous thrombosis than those without [98].

### NETs biomarkers and COVID-19-associated thromboembolism

Thromboembolism is common in COVID-19 patients [100] and independently associated with hospitalized mortality [101]. Immunostaining of lung, kidney, and heart tissues of COVID-19 patients revealed positive staining of H3Cit, MPO-DNA, NE, and DNA [102, 103]. Additionally, H3Cit, MPO, and DNA were colocalized with platelet and fibrin in blood vessels, indicating the involvement of NETs formation in the development of immunothrombosis [104].

The levels of plasma MPO-DNA and H3Cit were significantly higher in COVID-19 patients than healthy controls [105], and the level of plasma MPO-DNA positively correlated with the severity of COVID-19 [104]. Furthermore, the levels of plasma H3Cit-DNA, DNA, and NE correlated with those of widely recognized plasma markers for coagulation and fibrinolysis (i.e., D-dimer, TAT complex, and plasmin-antiplasmin) and endothelial activation and damage (i.e., VWF and ADAMTS13) [106]. The levels of plasma H3Cit and MPO-DNA were significantly higher in COVID-19 patients with VTE than those without. The areas under the curve of H3Cit and MPO-DNA for predicting VTE were 0.791 and 0.769, respectively [105]. The levels of serum H3Cit, MPO-DNA, DNA, and calprotectin were still higher in COVID-19 patients with both arterial thrombosis and VTE than those without thrombotic events, despite prophylactic anticoagulation was prescribed at the time of diagnosis of thrombotic events [107]. But such an association was not confirmed by a prospective cohort study, which demonstrated that the baseline level of plasma MPO-DNA could not predict the development of thrombosis [108] (Table 5).

**Table 5:** Studies evaluating NETs biomarkers in COVID-19 associated thromboembolism

First author/year	Study design	Included patients	Groups (No. patients)	Samples processing	NETs biomarkers	Analytical methods for NETs biomarkers	Detailed values
Ouwendijk et al (2021) [108]	Case–control and cohort	Critically ill patients with COVID-19	Thrombosis (*n* = 44) vs. No thrombosis (*n* = 33) vs. HC (*n* = 7)	Plasma	MPO-DNA	ELISA	NA
Petito et al (2021) [105]	Case–control and cohort	Hospitalized patients with COVID-19	VTE (*n* = 8) vs. No VTE (*n* = 27) vs. HC (*n* = 31)	Plasma, 4000 × g, 10 min	H3Cit	ELISA	NA
					MPO-DNA	ELISA	NA
Zuo et al (2021) [107]	Case–control	Hospitalized patients with COVID-19 and thrombosis	Thrombosis (*n* = 11) vs. No thrombosis (*n* = 33)	Serum	Calprotectin	ELISA	NA
					MPO-DNA	ELISA	NA
					H3Cit	ELISA	NA
					DNA	PicoGreen fluorimetry	NA

*Abbreviations:* *COVID-19* Coronavirus disease 2019, *ELISA* Enzyme-linked immunosorbent assay, *H3Cit* Citrullinated histone H3, *Min* Minute, *MPO* Myeloperoxidase, *NA* Not available, *NETs* Neutrophil extracellular traps, *VTE* Venous thromboembolism

### Potential therapeutic implications

NETs may be a potential therapeutic target for the management of thrombosis. First, DNase I can dissolve NETs structure, thereby compromising the formation of arterial thrombosis [109, 110], and reducing the weight of venous thrombus [42, 91] in mice. Ex vivo experiments measured the change of thrombus weight after thrombolysis of human PE, CAD, and IS thrombi and showed that either DNase I or tissue plasminogen activator (tPA) alone could induce thrombolysis [85], and a combination of DNase I and tPA further accelerated thrombolysis [45, 84, 85, 111]. This phenomenon may be attributed to the capacity of tPA to remove fibrin and that of DNase I to remove the "scaffold" of NETs connecting red blood cells and platelets [26]. Second, heparin, a frequently used anticoagulant, can remove histones in chromatin, then dismantle NETs [26]. Third, Cl-amidine, a PAD inhibitor, shows its ability to prevent thrombosis by inhibiting the NETs formation. Treatment with Cl-amidine can reduce the area of atherosclerotic lesion, prolong the time to carotid artery thrombosis in atherosclerosis mice [62], maintain the stability of cerebral perfusion, reduce the size of the ischemic lesion, and prevent from the development of thrombosis in IS mice [112]. GSK484, another potent and selective inhibitor of PAD4, strongly inhibits the NETs formation and thrombus deposition in mouse lungs [113]. Forth, ruxolitinib, a JAK1/JAK2 inhibitor, is a second-line drug for the treatment of MPN [114]. It can also abrogate the NETs formation and decrease the rate of stenosis-induced venous thrombosis in JAK2V617F-driven MPN mice [115]. Notably, all the above-mentioned evidence comes from animal and ex vivo experiments, and clinical studies of NETs as a therapeutic target for thrombosis have not been carried out yet.

### Limitations of current NETs biomarkers

Circulating NETs biomarkers include serum or plasma PAD4, H3Cit, MPO, NE, nucleosomes, or DNA, but their specificity of reflecting NETs formation remains uncertain. First, among the published studies, NETs biomarkers have been measured in human serum and plasma samples. However, it should be noted that neither serum nor plasma is the exact position of NETs formation. Second, PAD4 is not only involved in citrullination of histones during NETs formation, but also participates in other physiological processes, such as activation of vascular smooth muscle cells [116] and regulation of hematopoietic stem cell proliferation [117]. On the other hand, Cl-amidine, which has been widely used for the inhibition of PAD4 in NETs studies, is not a PAD4-specific inhibitor, but a pan-PAD inhibitor [118]. Third, citrullinated histones have been also observed during apoptosis [119]. Furthermore, in the absence of NETs-dependent stimulation, Western blot assay also shows positive expression of citrullinated histones in liver tissues [120]. Fourth, NE may be unnecessary for NETs formation, because NE deficiency or inhibition does not prevent NETs formation [121]. Fifth, MPO, which plays an important role in antimicrobial responses, is also expressed in monocytes and macrophages [122]. Sixth, nucleosomes may also originate from lymphocytes, red blood cells, and tumor cells, etc. [123]. Last, DNA can be either cell-free or bound with histones or other proteins in plasma and serum. Extracellular DNA is often considered a NETs biomarker, but can also be released during other cell death processes (i.e., apoptosis, necrosis, and pyroptosis) and active secretion (i.e., phagocytosis and egestion of DNA) [124]. Therefore, considering low specificity of a single NETs biomarker, it may be more reliable to combine two or more biomarkers for reflecting NETs formation.

Quantitative analyses of NETs biomarkers are clinically more useful and valuable. H3Cit, MPO-DNA, NE, and nucleosomes are often measured by enzyme-linked immunosorbent assay (ELISA), and DNA by quantitative polymerase chain reaction or fluorimetry assays. However, the type of samples, preanalytical sample preparation, and analytical methods used for measuring NETs biomarkers are heterogeneous among the published studies. First, plasma was employed for measuring NETs biomarkers in some studies, but serum in others. However, DNA levels are comparable in both plasma and serum of the same individuals [51]. Second, the methods on sample preparation, including the time from blood collection to sample processing, processing temperature, and centrifugal force, time, and frequency, often vary by study, which might influence experimental results. Prescriptive methods will helpfully improve the quality of samples and minimize preanalytical errors associated with sample preparation. Third, antibodies, assays, detection instruments, and manufacturers for detecting the same NETs biomarker are often diverse, thereby leading to the heterogeneity in experimental results among studies. Notably, the specificity of ELISA for the detection of some NETs biomarkers, such as the measurement of MPO-DNA complexes in human plasma, is questionable [125]. Therefore, robust, accurate, reproducible, well-standardized, and highly specific assays for measuring NETs biomarkers are required before drawing solid conclusions.

## Conclusion

**Fig. 2:**
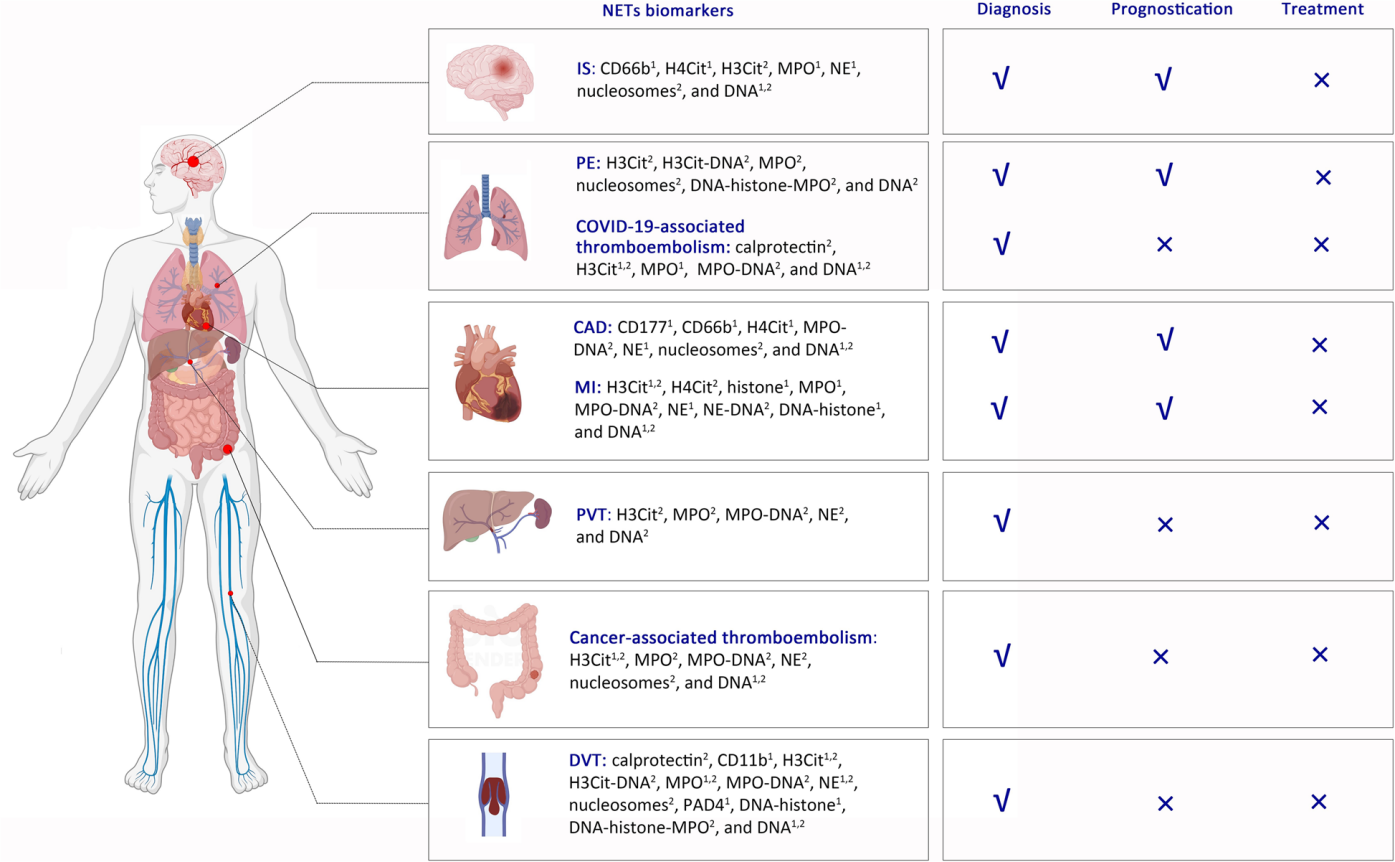
A schematic diagram of NETs biomarkers detected in human VTE, CAD, MI, IS, cancer-associated thromboembolism, and COVID-2019-associated thromboembolism ***Notes:*** ^1^ NETs biomarkers that have been explored in human thrombi specimens. ^2^ NETs biomarkers that have been explored in human plasma/serum. √ NETs biomarkers that have been explored for diagnosis, prognostication, and/or treatment. × NETs biomarkers that have not been explored for diagnosis, prognostication, or treatment. ***Abbreviations:*** CAD, Coronary artery diseases; COVID, Coronavirus disease 2019; DVT, Deep vein thrombosis; H3Cit, Citrullinated histone H3; H4Cit, Citrullinated histone H4; IS, Ischemic stroke; MI, Myocardial infarction; MPO, Myeloperoxidase; NE, Neutrophil elastase; NETs, Neutrophil extracellular traps; PAD4, Peptidyl arginine deiminase 4; PE, Pulmonary embolism; PVT, Portal vein thrombosis

Taken together, the effect of NETs formation on thrombosis is supported by a growing number of experimental and clinical studies, in which NETs biomarkers have been qualitatively and quantitatively measured. Particularly, H3Cit, MPO, MPO-DNA, NE, nucleosomes, and DNA, which are deemed as NETs biomarkers, have been evaluated in VTE, CAD, IS, cancer-associated thromboembolism, and COVID-19 associated thromboembolism (Fig. 2). Collectively, circulating NETs biomarkers seem to be associated with the presence and severity of thrombosis and correlate with hypercoagulability, but it remains unclear whether they can exactly reflect the NETs formation related to thrombosis, especially in patients with cancers and COVID-19. Instead of case–control or cross-sectional studies comparing between patients with thrombotic event and healthy population, cohort studies, where the development of a thrombotic event has been observed in the same population during follow up, should be more conductive in drawing more accurate and clinically relevant conclusions regarding diagnostic performance and predictive ability of NETs biomarkers. Routine detection of NETs biomarkers in patients with thrombosis cannot be considered until more robust evidence has been produced. Notably, it should be acknowledged that existing NETs biomarkers in serum and plasma and their detection methods are unsatisfactory. Besides, concomitant infection or inflammation, use of anticoagulants, antiplatelet drugs, and anti-cancer therapies, and effect of invasive or surgical procedures may influence the reliability of the current findings. In future, well-designed studies should also be necessary to clarify whether the change of NETs biomarkers is a cause or consequence of thrombosis by collecting blood samples before and after thrombosis.

## Data Availability

Not applicable.

## Abbreviations

ADAMTS13: A disintegrin and metalloproteinase with thrombospondin type-1 motif-13
AMI: Acute myocardial infarction
AIS: Acute ischemic stroke
CAD: Coronary artery diseases
CK: Creatine kinase
COVID-19: Coronavirus disease 2019
DVT: Deep vein thrombosis
ELISA: Enzyme-linked immunosorbent assay
HMGB1: High mobility group box 1
H3Cit: Citrullinated histone H3
H4Cit: Citrullinated histone H4
IS: Ischemic stroke
MACE: Major adverse cardiac events
MI: Myocardial infarction
MPN: Myeloproliferative neoplasms
MPO: Myeloperoxidase
NADPH: Nicotinamide adenine dinucleotide phosphate
NE: Neutrophil elastase
NETs: Neutrophil extracellular traps
OR: Odds ratio
PAD4: Peptidyl arginine deiminase 4
PCI: Percutaneous coronary intervention
PE: Pulmonary embolism
PVT: Portal vein thrombosis
ROS: Reactive oxygen species
STEMI: ST-elevation myocardial infarction
SVT: Splanchnic vein thrombosis
TAT: Thrombin-antithrombin
TLRs: Toll-like receptors
tPA: Tissue plasminogen activator
VTE: Venous thromboembolism

## References

[1] Furie B, Furie BC (2008). Mechanisms of thrombus formation [J]. N Engl J Med.

[2] Beckman MG, Hooper WC, Critchley SE, Ortel TL (2010). Venous thromboembolism: a public health concern [J]. Am J Prev Med.

[3] Roth GA, Johnson C, Abajobir A, Abd-Allah F, Abera SF, Abyu G (2017). Global, Regional, and National Burden of Cardiovascular Diseases for 10 Causes, 1990 to 2015 [J]. J Am Coll Cardiol.

[4] Lehman HK, Segal BH (2020). The role of neutrophils in host defense and disease [J]. J Allergy Clin Immunol.

[5] Thålin C, Hisada Y, Lundström S, Mackman N, Wallén H (2019). Neutrophil Extracellular Traps: Villains and Targets in Arterial, Venous, and Cancer-Associated Thrombosis [J]. Arterioscler Thromb Vasc Biol.

[6] Kapoor S, Opneja A, Nayak L (2018). The role of neutrophils in thrombosis [J]. Thromb Res.

[7] Kimball AS, Obi AT, Diaz JA, Henke PK (2016). The Emerging Role of NETs in Venous Thrombosis and Immunothrombosis [J]. Front Immunol.

[8] Moschonas IC, Tselepis AD (2019). The pathway of neutrophil extracellular traps towards atherosclerosis and thrombosis [J]. Atherosclerosis.

[9] Urban CF, Ermert D, Schmid M, Abu-Abed U, Goosmann C, Nacken W (2009). Neutrophil extracellular traps contain calprotectin, a cytosolic protein complex involved in host defense against Candida albicans [J]. PLoS Pathog.

[10] Brinkmann V, Reichard U, Goosmann C, Fauler B, Uhlemann Y, Weiss DS (2004). Neutrophil extracellular traps kill bacteria [J]. Science.

[11] Fuchs TA, Abed U, Goosmann C, Hurwitz R, Schulze I, Wahn V (2007). Novel cell death program leads to neutrophil extracellular traps [J]. J Cell Biol.

[12] Noubouossie DF, Reeves BN, Strahl BD, Key NS (2019). Neutrophils: back in the thrombosis spotlight [J]. Blood.

[13] Honda M, Kubes P (2018). Neutrophils and neutrophil extracellular traps in the liver and gastrointestinal system [J]. Nat Rev Gastroenterol Hepatol.

[14] Rohrbach AS, Slade DJ, Thompson PR, Mowen KA (2012). Activation of PAD4 in NET formation [J]. Front Immunol.

[15] Neeli I, Dwivedi N, Khan S, Radic M (2009). Regulation of extracellular chromatin release from neutrophils [J]. J Innate Immun.

[16] Lood C, Blanco LP, Purmalek MM, Carmona-Rivera C, De Ravin SS, Smith CK (2016). Neutrophil extracellular traps enriched in oxidized mitochondrial DNA are interferogenic and contribute to lupus-like disease [J]. Nat Med.

[17] Yousefi S, Mihalache C, Kozlowski E, Schmid I, Simon HU (2009). Viable neutrophils release mitochondrial DNA to form neutrophil extracellular traps [J]. Cell Death Differ.

[18] Wang Y, Li M, Stadler S, Correll S, Li P, Wang D (2009). Histone hypercitrullination mediates chromatin decondensation and neutrophil extracellular trap formation [J]. J Cell Biol.

[19] Silva CMS, Wanderley CWS, Veras FP, Sonego F, Nascimento DC, Gonçalves AV (2021). Gasdermin D inhibition prevents multiple organ dysfunction during sepsis by blocking NET formation [J]. Blood.

[20] Papayannopoulos V, Metzler KD, Hakkim A, Zychlinsky A (2010). Neutrophil elastase and myeloperoxidase regulate the formation of neutrophil extracellular traps [J]. J Cell Biol.

[21] Thiam HR, Wong SL, Qiu R, Kittisopikul M, Vahabikashi A, Goldman AE (2020). NETosis proceeds by cytoskeleton and endomembrane disassembly and PAD4-mediated chromatin decondensation and nuclear envelope rupture [J]. Proc Natl Acad Sci U S A.

[22] Li Y, Li M, Weigel B, Mall M, Werth VP, Liu ML (2020). Nuclear envelope rupture and NET formation is driven by PKCα-mediated lamin B disassembly [J]. EMBO Rep.

[23] Yipp BG, Kubes P (2013). NETosis: how vital is it? [J]. Blood.

[24] Remijsen Q, Vanden Berghe T, Wirawan E, Asselbergh B, Parthoens E, De Rycke R (2011). Neutrophil extracellular trap cell death requires both autophagy and superoxide generation [J]. Cell Res.

[25] Liang X, Liu L, Wang Y, Guo H, Fan H, Zhang C (2020). Autophagy-driven NETosis is a double-edged sword - Review [J]. Biomed Pharmacother.

[26] Fuchs TA, Brill A, Duerschmied D, Schatzberg D, Monestier M, Myers DD (2010). Extracellular DNA traps promote thrombosis [J]. Proc Natl Acad Sci U S A.

[27] Pircher J, Engelmann B, Massberg S, Schulz C (2019). Platelet-Neutrophil Crosstalk in Atherothrombosis [J]. Thromb Haemost.

[28] Maugeri N, Campana L, Gavina M, Covino C, De Metrio M, Panciroli C (2014). Activated platelets present high mobility group box 1 to neutrophils, inducing autophagy and promoting the extrusion of neutrophil extracellular traps [J]. J Thromb Haemost.

[29] Dyer MR, Chen Q, Haldeman S, Yazdani H, Hoffman R, Loughran P (2018). Deep vein thrombosis in mice is regulated by platelet HMGB1 through release of neutrophil-extracellular traps and DNA [J]. Sci Rep.

[30] Tadie JM, Bae HB, Jiang S, Park DW, Bell CP, Yang H (2013). HMGB1 promotes neutrophil extracellular trap formation through interactions with Toll-like receptor 4 [J]. Am J Physiol Lung Cell Mol Physiol.

[31] Stark K, Philippi V, Stockhausen S, Busse J, Antonelli A, Miller M (2016). Disulfide HMGB1 derived from platelets coordinates venous thrombosis in mice [J]. Blood.

[32] Yang X, Li L, Liu J, Lv B, Chen F (2016). Extracellular histones induce tissue factor expression in vascular endothelial cells via TLR and activation of NF-κB and AP-1 [J]. Thromb Res.

[33] Semeraro F, Ammollo CT, Morrissey JH, Dale GL, Friese P, Esmon NL (2011). Extracellular histones promote thrombin generation through platelet-dependent mechanisms: involvement of platelet TLR2 and TLR4 [J]. Blood.

[34] Fuchs TA, Bhandari AA, Wagner DD (2011). Histones induce rapid and profound thrombocytopenia in mice [J]. Blood.

[35] Ammollo CT, Semeraro F, Xu J, Esmon NL, Esmon CT (2011). Extracellular histones increase plasma thrombin generation by impairing thrombomodulin-dependent protein C activation [J]. J Thromb Haemost.

[36] Abrams ST, Su D, Sahraoui Y, Lin Z, Cheng Z, Nesbitt K (2021). Assembly of alternative prothrombinase by extracellular histones initiates and disseminates intravascular coagulation [J]. Blood.

[37] Pozzi N, Di Cera E (2016). Dual effect of histone H4 on prothrombin activation [J]. J Thromb Haemost.

[38] Semeraro F, Ammollo CT, Esmon NL, Esmon CT (2014). Histones induce phosphatidylserine exposure and a procoagulant phenotype in human red blood cells [J]. J Thromb Haemost.

[39] Varjú I, Longstaff C, Szabó L, Farkas ÁZ, Varga-Szabó VJ, Tanka-Salamon A (2015). DNA, histones and neutrophil extracellular traps exert anti-fibrinolytic effects in a plasma environment [J]. Thromb Haemost.

[40] Sorvillo N, Mizurini DM, Coxon C, Martinod K, Tilvawala R, Cherpokova D (2019). Plasma Peptidylarginine Deiminase IV Promotes VWF-Platelet String Formation and Accelerates Thrombosis After Vessel Injury [J]. Circ Res.

[41] Massberg S, Grahl L, von Bruehl ML, Manukyan D, Pfeiler S, Goosmann C (2010). Reciprocal coupling of coagulation and innate immunity via neutrophil serine proteases [J]. Nat Med.

[42] von Brühl ML, Stark K, Steinhart A, Chandraratne S, Konrad I, Lorenz M (2012). Monocytes, neutrophils, and platelets cooperate to initiate and propagate venous thrombosis in mice in vivo [J]. J Exp Med.

[43] Brill A, Fuchs TA, Savchenko AS, Thomas GM, Martinod K, De Meyer SF (2012). Neutrophil extracellular traps promote deep vein thrombosis in mice [J]. J Thromb Haemost.

[44] Sharma S, Hofbauer TM, Ondracek AS, Chausheva S, Alimohammadi A, Artner T (2021). Neutrophil extracellular traps promote fibrous vascular occlusions in chronic thrombosis [J]. Blood.

[45] Mangold A, Alias S, Scherz T, Hofbauer M, Jakowitsch J, Panzenböck A (2015). Coronary neutrophil extracellular trap burden and deoxyribonuclease activity in ST-elevation acute coronary syndrome are predictors of ST-segment resolution and infarct size [J]. Circ Res.

[46] Savchenko AS, Martinod K, Seidman MA, Wong SL, Borissoff JI, Piazza G (2014). Neutrophil extracellular traps form predominantly during the organizing stage of human venous thromboembolism development [J]. J Thromb Haemost.

[47] Arnalich F, Maldifassi MC, Ciria E, Codoceo R, Renart J, Fernández-Capitán C (2013). Plasma levels of mitochondrial and nuclear DNA in patients with massive pulmonary embolism in the emergency department: a prospective cohort study [J]. Crit Care.

[48] Diaz JA, Fuchs TA, Jackson TO, Kremer Hovinga JA, Lämmle B, Henke PK (2013). Plasma DNA is Elevated in Patients with Deep Vein Thrombosis [J]. J Vasc Surg Venous Lymphat Disord.

[49] van Montfoort ML, Stephan F, Lauw MN, Hutten BA, Van Mierlo GJ, Solati S (2013). Circulating nucleosomes and neutrophil activation as risk factors for deep vein thrombosis [J]. Arterioscler Thromb Vasc Biol.

[50] Jiménez-Alcázar M, Limacher A, Panda R, Méan M, Bitterling J, Peine S (2018). Circulating extracellular DNA is an independent predictor of mortality in elderly patients with venous thromboembolism [J]. PLoS ONE.

[51] Lee KH, Cavanaugh L, Leung H, Yan F, Ahmadi Z, Chong BH (2018). Quantification of NETs-associated markers by flow cytometry and serum assays in patients with thrombosis and sepsis [J]. Int J Lab Hematol.

[52] Martos L, Oto J, Fernández-Pardo Á, Plana E, Solmoirago MJ, Cana F (2020). Increase of Neutrophil Activation Markers in Venous Thrombosis-Contribution of Circulating Activated Protein C [J]. Int J Mol Sci.

[53] Medeiros SK, Emery B, Bhagirath V, Parpia S, Dwivedi DJ, Dwivedi NJ (2020). Does cell-free DNA promote coagulation and inhibit fibrinolysis in patients with unprovoked venous thromboembolism? [J]. Thromb Res.

[54] Ząbczyk M, Natorska J, Janion-Sadowska A, Metzgier-Gumiela A, Polak M, Plens K (2020). Prothrombotic fibrin clot properties associated with NETs formation characterize acute pulmonary embolism patients with higher mortality risk [J]. Sci Rep.

[55] Liu L, Zhang W, Su Y, Chen Y, Cao X, Wu J (2021). The impact of neutrophil extracellular traps on deep venous thrombosis in patients with traumatic fractures [J]. Clin Chim Acta.

[56] Turon F, Driever EG, Baiges A, Cerda E, García-Criado Á, Gilabert R (2021). Predicting portal thrombosis in cirrhosis: A prospective study of clinical, ultrasonographic and hemostatic factors [J]. J Hepatol.

[57] Xing Y, Jiang Y, Xing S, Mao T, Guan G, Niu Q (2022). Neutrophil extracellular traps are associated with enhanced procoagulant activity in liver cirrhosis patients with portal vein thrombosis [J]. J Clin Lab Anal.

[58] Smith P, Rosell A, Farm M, Bruzelius M, Aguilera Gatica K, Mackman N (2022). Markers of neutrophil activation and neutrophil extracellular traps in diagnosing patients with acute venous thromboembolism: A feasibility study based on two VTE cohorts [J]. PLoS ONE.

[59] Nordenholz KE, Mitchell AM, Kline JA (2008). Direct comparison of the diagnostic accuracy of fifty protein biological markers of pulmonary embolism for use in the emergency department [J]. Acad Emerg Med.

[60] Pugh RN, Murray-Lyon IM, Dawson JL, Pietroni MC, Williams R (1973). Transection of the oesophagus for bleeding oesophageal varices [J]. Br J Surg.

[61] Malakar AK, Choudhury D, Halder B, Paul P, Uddin A, Chakraborty S (2019). A review on coronary artery disease, its risk factors, and therapeutics [J]. J Cell Physiol.

[62] Knight JS, Luo W, O'Dell AA, Yalavarthi S, Zhao W, Subramanian V (2014). Peptidylarginine deiminase inhibition reduces vascular damage and modulates innate immune responses in murine models of atherosclerosis [J]. Circ Res.

[63] Warnatsch A, Ioannou M, Wang Q, Papayannopoulos V (2015). Inflammation. Neutrophil extracellular traps license macrophages for cytokine production in atherosclerosis [J]. Science.

[64] Liu Y, Carmona-Rivera C, Moore E, Seto NL, Knight JS, Pryor M (2018). Myeloid-Specific Deletion of Peptidylarginine Deiminase 4 Mitigates Atherosclerosis [J]. Front Immunol.

[65] Megens RT, Vijayan S, Lievens D, Döring Y, van Zandvoort MA, Grommes J (2012). Presence of luminal neutrophil extracellular traps in atherosclerosis [J]. Thromb Haemost.

[66] Franck G, Mawson TL, Folco EJ, Molinaro R, Ruvkun V, Engelbertsen D (2018). Roles of PAD4 and NETosis in Experimental Atherosclerosis and Arterial Injury: Implications for Superficial Erosion [J]. Circ Res.

[67] de Boer OJ, Li X, Teeling P, Mackaay C, Ploegmakers HJ, van der Loos CM (2013). Neutrophils, neutrophil extracellular traps and interleukin-17 associate with the organisation of thrombi in acute myocardial infarction [J]. Thromb Haemost.

[68] Stakos DA, Kambas K, Konstantinidis T, Mitroulis I, Apostolidou E, Arelaki S (2015). Expression of functional tissue factor by neutrophil extracellular traps in culprit artery of acute myocardial infarction [J]. Eur Heart J.

[69] Antonatos D, Patsilinakos S, Spanodimos S, Korkonikitas P, Tsigas D (2006). Cell-free DNA levels as a prognostic marker in acute myocardial infarction [J]. Ann N Y Acad Sci.

[70] Shimony A, Zahger D, Gilutz H, Goldstein H, Orlov G, Merkin M (2010). Cell free DNA detected by a novel method in acute ST-elevation myocardial infarction patients [J]. Acute Card Care.

[71] Borissoff JI, Joosen IA, Versteylen MO, Brill A, Fuchs TA, Savchenko AS (2013). Elevated levels of circulating DNA and chromatin are independently associated with severe coronary atherosclerosis and a prothrombotic state [J]. Arterioscler Thromb Vasc Biol.

[72] Cui M, Fan M, Jing R, Wang H, Qin J, Sheng H (2013). Cell-Free circulating DNA: a new biomarker for the acute coronary syndrome [J]. Cardiology.

[73] Langseth MS, Opstad TB, Bratseth V, Solheim S, Arnesen H, Pettersen A (2018). Markers of neutrophil extracellular traps are associated with adverse clinical outcome in stable coronary artery disease [J]. Eur J Prev Cardiol.

[74] Helseth R, Shetelig C, Andersen G, Langseth MS, Limalanathan S, Opstad TB (2019). Neutrophil Extracellular Trap Components Associate with Infarct Size, Ventricular Function, and Clinical Outcome in STEMI [J]. Mediators Inflamm.

[75] Lim HH, Jeong IH, An GD, Woo KS, Kim KH, Kim JM (2020). Evaluation of neutrophil extracellular traps as the circulating marker for patients with acute coronary syndrome and acute ischemic stroke [J]. J Clin Lab Anal.

[76] Liu J, Yang D, Wang X, Zhu Z, Wang T, Ma A (2019). Neutrophil extracellular traps and dsDNA predict outcomes among patients with ST-elevation myocardial infarction [J]. Sci Rep.

[77] Hofbauer TM, Mangold A, Scherz T, Seidl V, Panzenböck A, Ondracek AS (2019). Neutrophil extracellular traps and fibrocytes in ST-segment elevation myocardial infarction [J]. Basic Res Cardiol.

[78] Langseth MS, Helseth R, Ritschel V, Hansen CH, Andersen G, Eritsland J (2020). Double-Stranded DNA and NETs Components in Relation to Clinical Outcome After ST-Elevation Myocardial Infarction [J]. Sci Rep.

[79] Ramirez GA, Manfredi AA, Rovere-Querini P, Maugeri N (2016). Bet on NETs! Or on How to Translate Basic Science into Clinical Practice [J]. Front Immunol.

[80] Hally KE, Parker OM, Brunton-O'Sullivan MM, Harding SA, Larsen PD (2021). Linking Neutrophil Extracellular Traps and Platelet Activation: A Composite Biomarker Score for Predicting Outcomes after Acute Myocardial Infarction [J]. Thromb Haemost.

[81] Anderson JL, Morrow DA (2017). Acute Myocardial Infarction [J]. N Engl J Med.

[82] Maida CD, Norrito RL, Daidone M, Tuttolomondo A, Pinto A (2020). Neuroinflammatory Mechanisms in Ischemic Stroke: Focus on Cardioembolic Stroke, Background, and Therapeutic Approaches [J]. Int J Mol Sci.

[83] Boyko M, Ohayon S, Goldsmith T, Douvdevani A, Gruenbaum BF, Melamed I (2011). Cell-free DNA–a marker to predict ischemic brain damage in a rat stroke experimental model [J]. J Neurosurg Anesthesiol.

[84] Laridan E, Denorme F, Desender L, François O, Andersson T, Deckmyn H (2017). Neutrophil extracellular traps in ischemic stroke thrombi [J]. Ann Neurol.

[85] Ducroux C, Di Meglio L, Loyau S, Delbosc S, Boisseau W, Deschildre C (2018). Thrombus Neutrophil Extracellular Traps Content Impair tPA-Induced Thrombolysis in Acute Ischemic Stroke [J]. Stroke.

[86] O'Connell GC, Petrone AB, Tennant CS, Lucke-Wold N, Kabbani Y, Tarabishy AR (2017). Circulating extracellular DNA levels are acutely elevated in ischaemic stroke and associated with innate immune system activation [J]. Brain Inj.

[87] Vallés J, Lago A, Santos MT, Latorre AM, Tembl JI, Salom JB (2017). Neutrophil extracellular traps are increased in patients with acute ischemic stroke: prognostic significance [J]. Thromb Haemost.

[88] Thålin C, Demers M, Blomgren B, Wong SL, von Arbin M, von Heijne A (2016). NETosis promotes cancer-associated arterial microthrombosis presenting as ischemic stroke with troponin elevation [J]. Thromb Res.

[89] Tuzovic M, Herrmann J, Iliescu C, Marmagkiolis K, Ziaeian B, Yang EH (2018). Arterial Thrombosis in Patients with Cancer [J]. Curr Treat Options Cardiovasc Med.

[90] Ay C, Pabinger I, Cohen AT (2017). Cancer-associated venous thromboembolism: Burden, mechanisms, and management [J]. Thromb Haemost.

[91] Hisada Y, Grover SP, Maqsood A, Houston R, Ay C, Noubouossie DF (2020). Neutrophils and neutrophil extracellular traps enhance venous thrombosis in mice bearing human pancreatic tumors [J]. Haematologica.

[92] Demers M, Krause DS, Schatzberg D, Martinod K, Voorhees JR, Fuchs TA (2012). Cancers predispose neutrophils to release extracellular DNA traps that contribute to cancer-associated thrombosis [J]. Proc Natl Acad Sci U S A.

[93] Yang C, Sun W, Cui W, Li X, Yao J, Jia X (2015). Procoagulant role of neutrophil extracellular traps in patients with gastric cancer [J]. Int J Clin Exp Pathol.

[94] Mauracher LM, Posch F, Martinod K, Grilz E, Däullary T, Hell L (2018). Citrullinated histone H3, a biomarker of neutrophil extracellular trap formation, predicts the risk of venous thromboembolism in cancer patients [J]. J Thromb Haemost.

[95] Bang OY, Chung JW, Cho YH, Oh MJ, Seo WK, Kim GM (2019). Circulating DNAs, a Marker of Neutrophil Extracellular Traposis and Cancer-Related Stroke: The OASIS-Cancer Study [J]. Stroke.

[96] Grilz E, Mauracher LM, Posch F, Königsbrügge O, Zöchbauer-Müller S, Marosi C (2019). Citrullinated histone H3, a biomarker for neutrophil extracellular trap formation, predicts the risk of mortality in patients with cancer [J]. Br J Haematol.

[97] Seo JD, Gu JY, Jung HS, Kim YJ, Kim HK (2019). Contact System Activation and Neutrophil Extracellular Trap Markers: Risk Factors for Portal Vein Thrombosis in Patients With Hepatocellular Carcinoma [J]. Clin Appl Thromb Hemost.

[98] Guy A, Favre S, Labrouche-Colomer S, Deloison L, Gourdou-Latyszenok V, Renault MA (2019). High circulating levels of MPO-DNA are associated with thrombosis in patients with MPN [J]. Leukemia.

[99] Zhang Y, Wang C, Yu M, Zhao X, Du J, Li Y (2019). Neutrophil extracellular traps induced by activated platelets contribute to procoagulant activity in patients with colorectal cancer [J]. Thromb Res.

[100] Klok FA, Kruip M, van der Meer NJM, Arbous MS, Gommers D, Kant KM (2020). Confirmation of the high cumulative incidence of thrombotic complications in critically ill ICU patients with COVID-19: An updated analysis [J]. Thromb Res.

[101] Bilaloglu S, Aphinyanaphongs Y, Jones S, Iturrate E, Hochman J, Berger JS (2020). Thrombosis in Hospitalized Patients With COVID-19 in a New York City Health System [J]. JAMA.

[102] Leppkes M, Knopf J, Naschberger E, Lindemann A, Singh J, Herrmann I (2020). Vascular occlusion by neutrophil extracellular traps in COVID-19 [J]. EBioMedicine.

[103] Nicolai L, Leunig A, Brambs S, Kaiser R, Weinberger T, Weigand M (2020). Immunothrombotic Dysregulation in COVID-19 Pneumonia Is Associated With Respiratory Failure and Coagulopathy [J]. Circulation.

[104] Middleton EA, He XY, Denorme F, Campbell RA, Ng D, Salvatore SP (2020). Neutrophil extracellular traps contribute to immunothrombosis in COVID-19 acute respiratory distress syndrome [J]. Blood.

[105] Petito E, Falcinelli E, Paliani U, Cesari E, Vaudo G, Sebastiano M (2021). Association of Neutrophil Activation, More Than Platelet Activation, With Thrombotic Complications in Coronavirus Disease 2019 [J]. J Infect Dis.

[106] Ng H, Havervall S, Rosell A, Aguilera K, Parv K, von Meijenfeldt FA (2021). Circulating Markers of Neutrophil Extracellular Traps Are of Prognostic Value in Patients With COVID-19 [J]. Arterioscler Thromb Vasc Biol.

[107] Zuo Y, Zuo M, Yalavarthi S, Gockman K, Madison JA, Shi H (2021). Neutrophil extracellular traps and thrombosis in COVID-19 [J]. J Thromb Thrombolysis.

[108] Ouwendijk WJD, Raadsen MP, van Kampen JJA, Verdijk RM, von der Thusen JH, Guo L (2021). High Levels of Neutrophil Extracellular Traps Persist in the Lower Respiratory Tract of Critically Ill Patients With Coronavirus Disease 2019 [J]. J Infect Dis.

[109] Arroyo AB, Fernández-Pérez MP, Del Monte A, Águila S, Méndez R, Hernández-Antolín R (2021). miR-146a is a pivotal regulator of neutrophil extracellular trap formation promoting thrombosis [J]. Haematologica.

[110] Leal AC, Mizurini DM, Gomes T, Rochael NC, Saraiva EM, Dias MS (2017). Tumor-Derived Exosomes Induce the Formation of Neutrophil Extracellular Traps: Implications For The Establishment of Cancer-Associated Thrombosis [J]. Sci Rep.

[111] Li T, Peng R, Wang F, Hua L, Liu S, Han Z (2020). Lysophosphatidic acid promotes thrombus stability by inducing rapid formation of neutrophil extracellular traps: A new mechanism of thrombosis [J]. J Thromb Haemost.

[112] Peña-Martínez C, Durán-Laforet V, García-Culebras A, Ostos F, Hernández-Jiménez M, Bravo-Ferrer I (2019). Pharmacological Modulation of Neutrophil Extracellular Traps Reverses Thrombotic Stroke tPA (Tissue-Type Plasminogen Activator) Resistance [J]. Stroke.

[113] Perdomo J, Leung HHL, Ahmadi Z, Yan F, Chong JJH, Passam FH (2019). Neutrophil activation and NETosis are the major drivers of thrombosis in heparin-induced thrombocytopenia [J]. Nat Commun.

[114] Finazzi G, De Stefano V, Barbui T (2018). Splanchnic vein thrombosis in myeloproliferative neoplasms: treatment algorithm 2018 [J]. Blood Cancer J.

[115] Wolach O, Sellar RS, Martinod K, Cherpokova D, McConkey M, Chappell RJ (2018). Increased neutrophil extracellular trap formation promotes thrombosis in myeloproliferative neoplasms [J]. Sci Transl Med.

[116] Park B, Yim JH, Lee HK, Kim BO, Pyo S (2015). Ramalin inhibits VCAM-1 expression and adhesion of monocyte to vascular smooth muscle cells through MAPK and PADI4-dependent NF-kB and AP-1 pathways [J]. Biosci Biotechnol Biochem.

[117] Nakashima K, Arai S, Suzuki A, Nariai Y, Urano T, Nakayama M (2013). PAD4 regulates proliferation of multipotent haematopoietic cells by controlling c-myc expression [J]. Nat Commun.

[118] Mondal S, Thompson PR (2019). Protein Arginine Deiminases (PADs): Biochemistry and Chemical Biology of Protein Citrullination [J]. Acc Chem Res.

[119] Tanikawa C, Espinosa M, Suzuki A, Masuda K, Yamamoto K, Tsuchiya E (2012). Regulation of histone modification and chromatin structure by the p53-PADI4 pathway [J]. Nat Commun.

[120] Zhao X, Yang L, Chang N, Hou L, Zhou X, Yang L (2020). Neutrophils undergo switch of apoptosis to NETosis during murine fatty liver injury via S1P receptor 2 signaling [J]. Cell Death Dis.

[121] Martinod K, Witsch T, Farley K, Gallant M, Remold-O'Donnell E, Wagner DD (2016). Neutrophil elastase-deficient mice form neutrophil extracellular traps in an experimental model of deep vein thrombosis [J]. J Thromb Haemost.

[122] Prokopowicz Z, Marcinkiewicz J, Katz DR, Chain BM (2012). Neutrophil myeloperoxidase: soldier and statesman [J]. Arch Immunol Ther Exp (Warsz).

[123] Holdenrieder S, Stieber P (2009). Clinical use of circulating nucleosomes [J]. Crit Rev Clin Lab Sci.

[124] Kustanovich A, Schwartz R, Peretz T, Grinshpun A (2019). Life and death of circulating cell-free DNA [J]. Cancer Biol Ther.

[125] Hayden H, Ibrahim N, Klopf J, Zagrapan B, Mauracher LM, Hell L (2021). ELISA detection of MPO-DNA complexes in human plasma is error-prone and yields limited information on neutrophil extracellular traps formed in vivo [J]. PLoS ONE.

